# Characteristics and Resistance Patterns of Gram-Negative Bacterial Isolates to Third-Generation Cephalosporins in Oncology ICU Patients: A Ten-Year Retrospective Analysis

**DOI:** 10.3390/pathogens15101083

**Published:** 2026-10-15

**Authors:** Omar Khresat, Sawsan Mubarak, Joud Jarrah, Hadeel AlGhawrie

**Affiliations:** 1 Infection Control Program, King Hussein Cancer Center, Amman 11941, Jordan; okhresat@khcc.jo (O.K.); smubarak@khcc.jo (S.M.); 2 Department of Medicine, King Hussein Cancer Center, Amman 11941, Jordan; jj.11090@khcc.jo

**Keywords:** antimicrobial resistance, third-generation cephalosporins, oncology, intensive care unit, gram-negative bacteria, hematological malignancy

## Abstract

Third-generation cephalosporin (3GC) resistance threatens empirical therapy in vulnerable critically ill patients. We conducted a decade-long single-center retrospective study of 3528 Gram-negative isolates from 1946 adult and pediatric oncology intensive care unit (ICU) patients between 2015 and 2024 at King Hussein Cancer Center. This study aims to characterize 3GS resistance in oncology ICU patients. The 3GC resistance was 59.2% among *Enterobacterales* and other fermenting organisms (evaluated against the full three-agent panel) and 67.2%among non-fermenters (*Pseudomonas aeruginosa*, *Acinetobacter* spp., *Stenotrophomonas maltophilia*; evaluated against a narrower, organism-appropriate agent subset); these two estimates are not directly comparable given the differing susceptibility panels tested. Resistance was highest among the *Acinetobacter calcoaceticus-baumannii* complex (96.9%), followed by *Escherichia coli* (70.5%), *Enterobacter cloacae* (65.5%), and *Klebsiella pneumoniae* (59.6%). In unadjusted patient-level analyses, resistance was significantly higher among patients with hematological malignancy than solid tumors (73.1% vs. 57.8%; OR 1.98, 95% CI 1.59–2.48; *p* < 0.001) and among pediatric versus adult ICU isolates (70.8% vs. 60.9%; OR 1.55, 95% CI 1.11–2.18; *p* = 0.010). Seven-day mortality did not differ by resistance status (44.1% vs. 41.6%; unadjusted OR 1.11, 95% CI 0.92–1.33). The 3GC resistance varies by organism, malignancy type and ICU population in unadjusted analyses, supporting population-specific antibiograms and stronger stewardship.

## 1. Introduction

Multidrug-resistant organisms (MDROs) threaten many areas of medicine, including cancer therapy, intensive care unit (ICU) care, solid organ transplantation and invasive surgery. Resistant Gram-negative organisms contribute substantially to the global burden of antimicrobial resistance [1,2]. Specific Gram-negative species have been named as critical- or high-priority pathogens by the World Health Organization based on two key criteria: ability to cause severe infections that are difficult to treat and a very limited number of drugs in development to treat these infections [3,4]. Third-generation cephalosporins (3GCs) (in this study, ceftazidime, ceftriaxone, and cefixime) are among the antimicrobial classes most impacted by antimicrobial resistance (AMR). The 3GCs were frequently used as empirical therapy for many severe Gram-negative infections for decades due to broad coverage, safety, and administration convenience. However, resistance to 3GCs has increased over time as a result of production of extended-spectrum beta-lactamases (ESBLs), plasmid-mediated AmpC beta-lactamases, and chromosomally mediated changes including porin channel loss and overexpression of efflux pumps. Resistance to 3GCs is often accompanied by resistance or decreased susceptibility to fluoroquinolones and aminoglycosides, which further limits treatment options and has led to the increased use of broader-spectrum antibiotics, which may be more toxic or expensive [5,6].

Resistance has more serious consequences in patients with cancer, who are inherently susceptible to infection because of their malignancy and its treatment. Neutropenia, mucosal damage and immunosuppression after hematopoietic stem cell transplantation all weaken the body’s physical barriers against infection and may prolong susceptibility [7,8,9]. In addition, many cancer patients experience longer durations of hospitalization (including stays in intensive care units), are frequently exposed to central venous catheters and other invasive devices, and often receive repeated courses of antibiotics (usually broad-spectrum) [8,9,10]. For these reasons, colonization with resistant bacteria and progression to invasive infection are of greater concern in patients with cancer [10,11].

Clinicians treating neutropenic or critically ill patients with cancer are thus faced with an ongoing therapeutic conundrum. Associations between delayed or inappropriate initial antimicrobial administration and negative clinical outcomes have been well established in this patient population, lending heavy inertia toward more expansive initial coverage [12,13]. However, unnecessarily broadening therapy to include carbapenems or other last-line agents may contribute to further antimicrobial selective pressure, leading to emergence of highly resistant pathogens such as carbapenem-resistant *Enterobacterales* and few, if any, drugs left to treat them [12,13]. Balancing timely empirical coverage against judicious stewardship therefore requires unit-specific susceptibility data reflecting the local epidemiology of the population being treated, rather than generic treatment algorithms [12,13].

Cumulative antibiograms address this need by summarizing local laboratory susceptibility data to directly inform empiric prescribing and stewardship programs [14,15]. Their utility depends on consistent methodology, as differences in organism identification, breakpoint interpretation, and duplicate-isolate handling can all distort calculated resistance rates [14,15,16]. These effects are magnified over longer surveillance periods, particularly given periodic CLSI breakpoint revisions [15,16]. Although this has been recognized as a worldwide threat, descriptions of Gram-negative resistance patterns unique to the ICU setting in oncology patients are lacking, especially in the Middle East. To our knowledge, there have been few published studies at the institutional level examining longitudinal antimicrobial resistance patterns. While several studies have evaluated the prevalence of resistant organisms within the region, they have been limited by short duration or a multicenter design. Thus, there is a lack of evidence examining changes in antimicrobial resistance patterns over time in a single institution that services exclusively an oncology critical care population including both adult and pediatric patients [17,18].

The aim of this study was to characterize third-generation cephalosporin (3GC) resistance among Gram-negative isolates from adult and pediatric oncology ICU patients at a single comprehensive cancer center over a ten-year period (2015–2024), in order to inform locally tailored antimicrobial stewardship and empirical therapy. Specifically, we estimated the overall prevalence and annual trend of resistance, described resistance by organism and specimen source, and explored unadjusted associations between resistance and patient characteristics, and between resistance and seven-day mortality.

## 2. Materials and Methods

**Figure 1:**
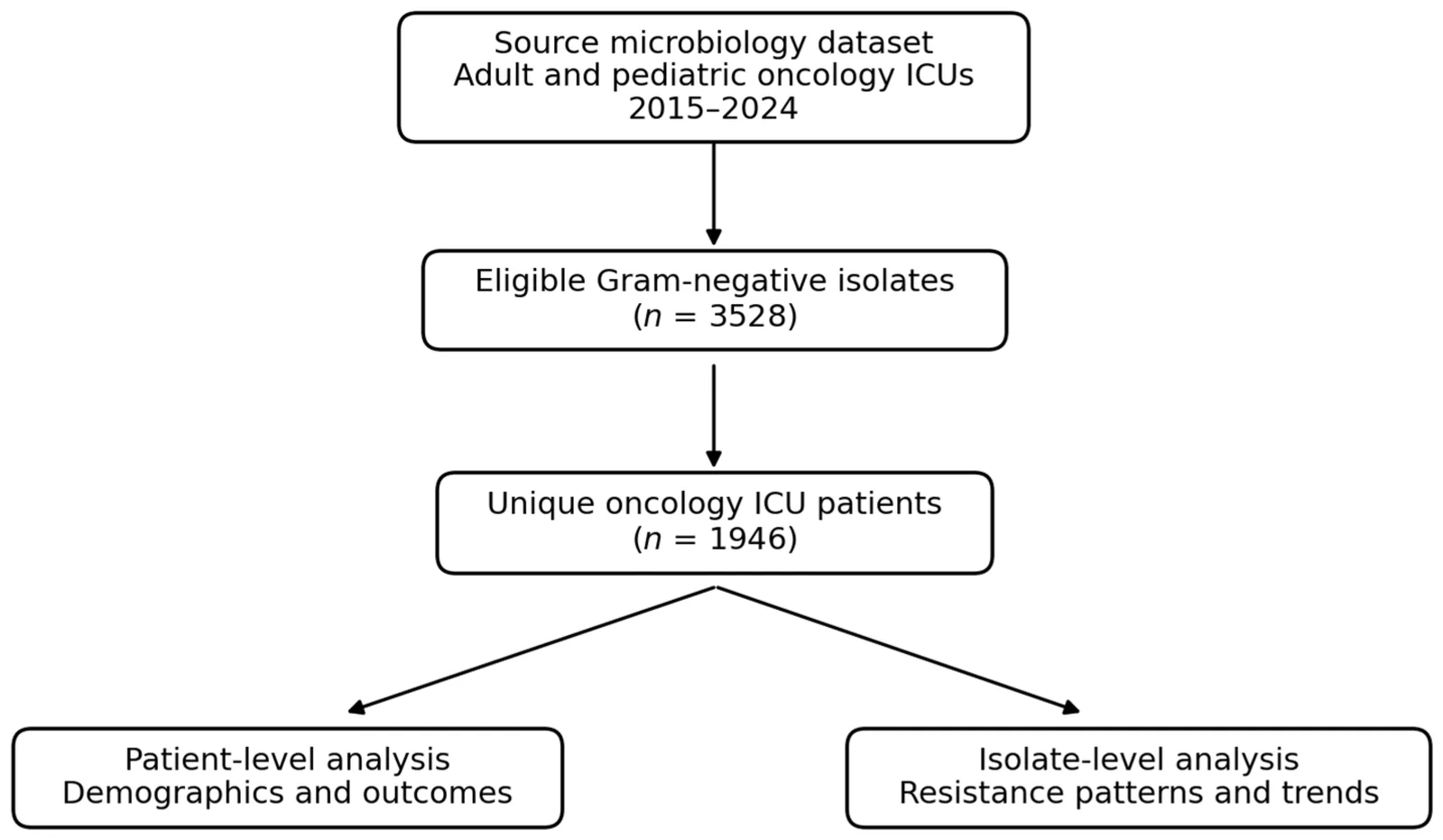
Study flow and analytical populations.

This retrospective observational study of routinely collected microbiology and clinically linked data was conducted over a ten-year period (1 January 2015–31 December 2024) to characterize third-generation cephalosporin (3GC) resistance among Gram-negative isolates from adult and pediatric oncology ICU patients. Two analytical units were defined: individual patients, for demographic, outcome and association analyses, and bacterial isolates, for descriptive susceptibility analyses (organism, specimen source and year; Figure 1).

Third-generation cephalosporin resistance was defined at the isolate level using antimicrobial susceptibility results for ceftazidime, ceftriaxone, and cefixime, interpreted using CLSI M100 breakpoints [16]. Susceptibility testing was performed using the VITEK 2 automated system (bioMérieux, Marcy-l’Étoile, France), whose onboard expert system applies organism-specific CLSI breakpoints according to the software and card version in use. Each isolate was interpreted with the M100 edition in effect at the time of testing (M100-S25 in 2015 through the 34th edition in 2024), as applied by the VITEK 2 software. Only final categorical results (S/I/R) were retained; MIC values were not available and isolates were not reinterpreted using a single current edition. The edition and VITEK 2 software/card version applied in each year are listed in Table S1. Isolates were classified as susceptible or non-susceptible. For the primary outcome, an “intermediate” result for a tested 3GC agent was grouped with “resistant”, a conservative non-susceptibility definition commonly used in antibiogram reporting. An isolate was classified as 3GC-resistant if it was resistant (or intermediate) to all 3GC agents tested for that isolate; isolates susceptible to at least one tested 3GC agent were classified as 3GC-susceptible. Not all three agents were tested against every isolate, reflecting routine laboratory testing panels that vary by organism and specimen type; the resistance determination was therefore based on the full complement of 3GC agents actually tested for each individual isolate, rather than requiring uniform testing of all three agents across all isolates. Because ceftriaxone and cefixime are not clinically active against, and are not routinely tested for, non-fermenting Gram-negative organisms (*Pseudomonas aeruginosa*, *Acinetobacter* spp.), the 3GC panel actually evaluated differed systematically by organism group: *Enterobacterales* and other fermenters were tested against ceftazidime, ceftriaxone, and cefixime (≥97% testing completeness for each agent), *P. aeruginosa* was tested against ceftazidime only, and *Acinetobacter* spp. was tested against ceftazidime and ceftriaxone only. Consequently, all 3GC resistance results in this manuscript are stratified by fermenter status (*Enterobacterales*/other fermenters vs. non-fermenters) rather than reported solely as a single pooled estimate, and readers should note that “3GC resistance” among non-fermenters reflects a narrower, organism-appropriate agent subset rather than the complete three-agent panel applied to *Enterobacterales*. Seven-day all-cause mortality was defined as death within seven days of the date of the first eligible culture. For patient-level analyses, each patient’s resistance status was that of their first eligible isolate.

The analysis had two components: a patient-level cohort (age, sex, malignancy type, ICU location, resistance status, mortality) and an isolate-level cohort (organism distribution, specimen source, susceptibility, annual trends).

This retrospective cohort study was conducted at King Hussein Cancer Center (KHCC) in Amman, Jordan. KHCC is a comprehensive cancer center that offers medical oncology and hematopoietic stem cell transplant services for adults and children. Participants included all patients treated at KHCC’s adult ICU or pediatric ICU who had a documented eligible Gram-negative isolate during the study period.

Isolate ascertainment in this study followed a laboratory-based surveillance approach methodologically analogous to the U.S. Centers for Disease Control and Prevention’s National Healthcare Safety Network (NHSN) “Laboratory-Identified (LabID) Event” reporting framework, in which laboratory testing data from routine clinical cultures are used without prospective, patient-level clinical adjudication of infection status. Accordingly, isolates in the present study represent true infection, colonization, or clinically indeterminate significance, and this distinction is not systematically captured in the microbiology laboratory information system; isolates are therefore referred to throughout as “Gram-negative isolates” rather than as confirmed infections.

Duplicate isolates were excluded using a laboratory-based rule: an isolate was retained if it was the first isolate for a given patient, organism and specimen source within a calendar month. This rule is less restrictive than the CLSI M39 convention (first isolate per patient per organism per analysis period, irrespective of specimen source). A patient could therefore contribute more than one isolate (mean 1.8 per patient), including repeat isolates of the same organism and source in successive months. Because persistently colonized, long-stay patients may contribute repeat resistant isolates, isolate-level prevalence may be overestimated. Clinical data abstraction included the microbiology laboratory information system (organism, specimen, date, susceptibility); clinical/ administrative databases (age, sex, malignancy category, ICU type, seven-day mortality); and calculated variables (study year, resistance category). These sources were merged into a single analytic dataset at the isolate level. Isolates with missing susceptibility results for a given 3GC agent were not imputed; consistent with standard antibiogram methodology, the 3GC resistance classification for each isolate was instead based only on the agents actually tested for that isolate, as described above.

Primary Epidemiological Measure: 3GC resistance prevalence (%) = (Number of resistant isolates (as defined above)/Total eligible isolates) × 100, calculated overall and by organism, specimen, and year [14,15].

Analyses were performed to identify missing values, miscoding, and outliers. Patient- and isolate-level denominators were maintained. Continuous variables were summarized with mean ± SD or median (IQR), as appropriate. Categorical variables were summarized with count (%). Prevalence was computed using isolates as the unit of analysis. Because isolates from the same patient are correlated, we handled clustering at three levels. (i) Isolate-level proportions, confidence intervals and *p*-values (overall, by organism, specimen source and year, including the chi-square and Cochran–Armitage tests) treat isolates as independent, are likely too optimistic, and are presented as descriptive. (ii) Overall prevalence was re-estimated at the patient level using one resistance-status observation per patient (first eligible isolate; Wald 95% CI), and we treat this as the more appropriate measure of uncertainty. (iii) All association and mortality analyses (malignancy type, ICU setting, sex, age, seven-day mortality) were performed at the patient level, so no patient contributed more than once.

Associations between patient characteristics (sex, malignancy category, ICU type) and resistance, and between resistance status and seven-day mortality, were assessed at the patient level (one observation per patient) using chi-square tests and unadjusted odds ratios (Wald 95% CI). Age was compared using the Mann–Whitney U test. Year was assessed as described above. No multivariable models were fitted; all associations are unadjusted. Data on severity of illness, neutropenia, infection versus colonization and antimicrobial therapy were not available. No adjustment was made for multiple comparisons, and results are exploratory and hypothesis-generating. Statistical analyses were performed using IBM SPSS Statistics, version 32 (IBM Corp., Armonk, NY, USA).

## 3. Results

### 3.1. Patient Characteristics

A total of 1946 adult and pediatric oncology ICU patients met eligibility criteria over the ten-year study period. The mean patient age was 53.47 ± 20.07 years (range 1–95 years). Males comprised the majority of the cohort (1104/1946, 56.7%), and most patients were admitted to the adult ICU (1768/1946, 90.9%) rather than the pediatric ICU (178/1946, 9.1%). Solid tumors were the predominant malignancy type (1440/1946, 74.0%), followed by hematological malignancies (506/1946, 26.0%). Seven-day all-cause mortality was recorded in 839 of 1946 patients (43.1%) as illustrated in Table 1.

### 3.2. Overall Resistance Burden

Among 3528 eligible Gram-negative isolates, 2187 demonstrated resistance, whereas 1341 were susceptible to third-generation cephalosporins. The overall prevalence of resistance was 62.0% (95% CI 60.4–63.6%). This interval treats the 3528 isolates as independent, although they were derived from 1946 patients (mean 1.8 isolates per patient), and it therefore likely understates uncertainty. Using one observation per patient (first eligible isolate), resistance prevalence was 61.8% (1203/1946; 95% CI 59.7–64.0%), similar to the isolate-level estimate (62.0%; 95% CI 60.4–63.6%) but with a wider interval, which we consider the more appropriate expression of uncertainty.

### 3.3. Organism Distribution

Thirty-seven distinct Gram-negative species/groups were identified among the 3528 eligible isolates. *Escherichia coli* was the most common (1077/3528, 30.5%), followed by *Klebsiella pneumoniae* (691/3528, 19.6%), the *Acinetobacter calcoaceticus-baumannii* complex (607/3528, 17.2%), and *Pseudomonas aeruginosa* (588/3528, 16.7%); together these four organisms accounted for 2963/3528 isolates (84.0%). The remaining 33 species/groups each represented <4% of isolates (Table 2).

### 3.4. Resistance by Organism

**Figure 2:**
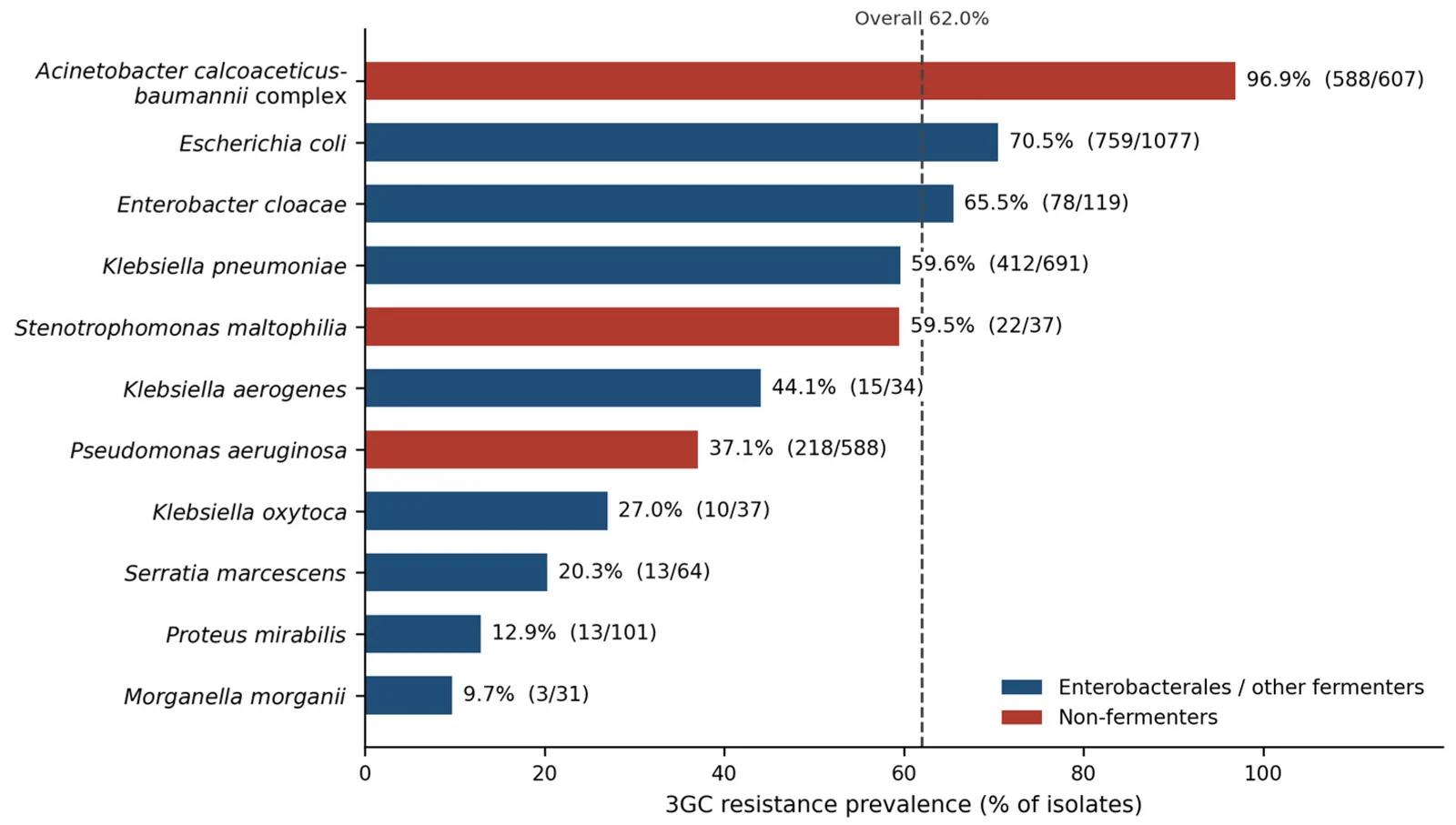
Third-generation cephalosporin resistance by species among Gram-negative isolates.

Resistance varied widely by species (Table 2, Figure 2). The *Acinetobacter calcoaceticus-baumannii* complex showed near-universal resistance (588/607, 96.9%), followed by *Escherichia coli* (759/1077, 70.5%), *Enterobacter cloacae* (78/119, 65.5%) and *Klebsiella pneumoniae* (412/691, 59.6%). *Pseudomonas aeruginosa* showed lower resistance (218/588, 37.1%), assessed against ceftazidime alone. Resistance was lowest among *Morganella morganii* (9.7%), *Proteus mirabilis* (12.9%) and *Serratia marcescens* (20.3%), although *M. morganii* and several other species were represented by few isolates (*n* = 31–101). Exact prevalence and isolate counts for all species are shown in Figure 2.

### 3.5. Resistance by Specimen Source

Isolates were most frequently recovered from tracheal aspirates (Trap, *n* = 1369), urine (*n* = 649), and blood (*n* = 406), which together accounted for the majority of specimens. Resistance varied by specimen source; blood isolates, the category most directly linked to bacteremia, showed a resistance prevalence of 70.0%, as illustrated in Table 3.

### 3.6. Annual Trend

**Figure 3:**
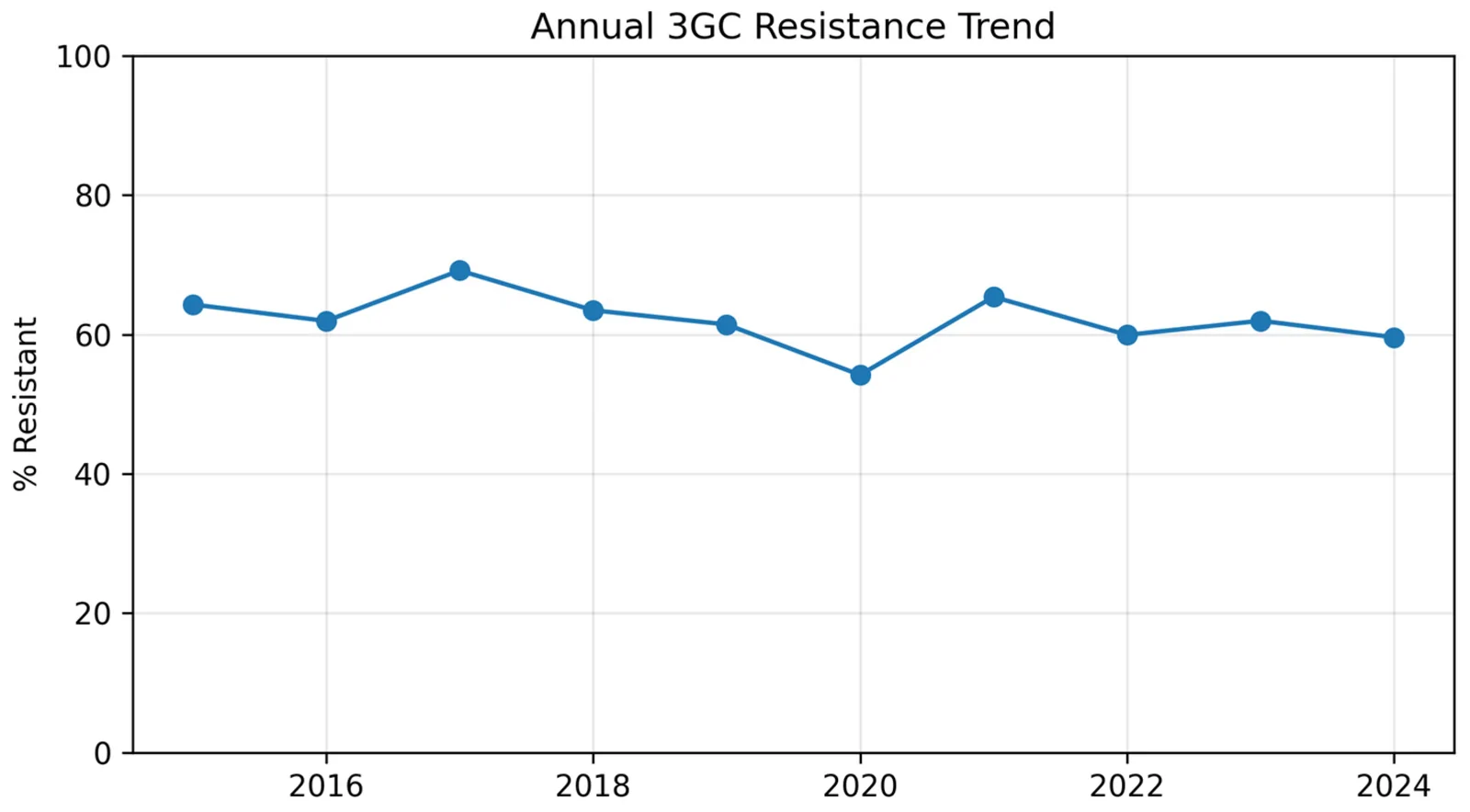
Annual third-generation cephalosporin resistance prevalence, 2015–2024.

Resistance remained consistently above 50% each year (Figure 3). The lowest annual prevalence was 54.2% in 2020, while the highest was 69.2% in 2017. Resistance prevalence differed significantly between years (chi-square *p* = 0.012), but this omnibus test does not indicate direction. A Cochran-Armitage test for linear trend showed only a modest, non-statistically significant downward trend across the study period (Z = −1.88, *p* = 0.060; odds ratio per year 0.977, 95% CI 0.954–1.001). Taken together, these results indicate that 3GC resistance fluctuated from year to year without a statistically significant sustained increase or decline over the decade. Both tests treat isolates as independent; with within-patient correlation, the *p*-values (0.012 and 0.060) are likely too small, so the finding of year-to-year heterogeneity should be considered exploratory.

### 3.7. Resistance to Other Antimicrobial Agents

Resistance to other commonly tested antimicrobial agents was generally lower than that observed for 3GCs but remained substantial for several agents (Table 4). Ceftriaxone (61.3%), ciprofloxacin (58.1%), and cefixime (58.1%) showed the highest resistance rates, while amikacin (24.7%), meropenem (26.3%), imipenem (26.7%), and ertapenem (12.7%) demonstrated comparatively preserved susceptibility. Piperacillin-tazobactam and gentamicin showed intermediate resistance levels (35.5% and 37.3%, respectively).

### 3.8. Factors Associated with Resistance

**Figure 4:**
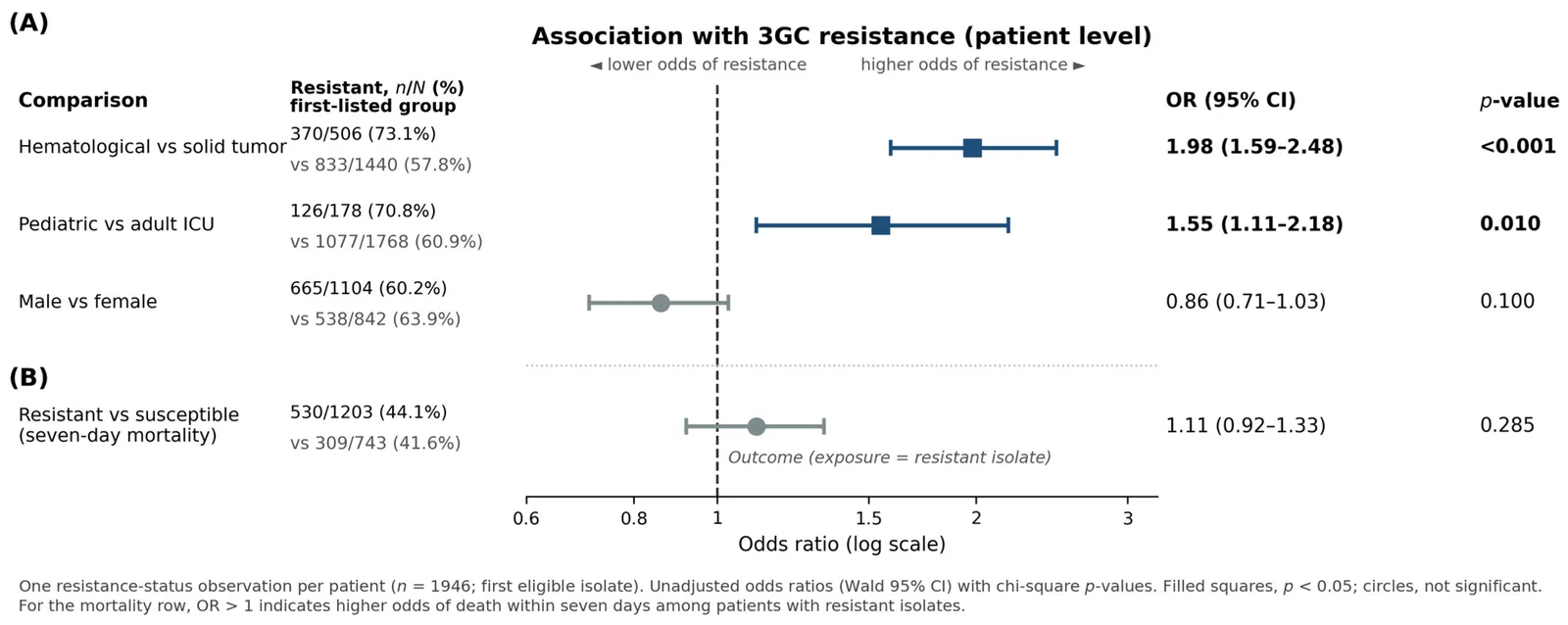
Unadjusted patient-level associations (*n* = 1946; one observation per patient, first eligible isolate). (**A**) Odds of 3GC resistance by patient characteristic. (**B**) Odds of seven-day mortality in patients with resistant vs. susceptible isolates (OR > 1 = higher odds of death with resistance). Wald 95% CI; chi-square *p*-values.

Resistance was significantly associated with malignancy type and ICU setting at the patient level. Patients with hematological malignancy showed higher resistance prevalence than those with solid tumors (370/506, 73.1% vs. 833/1440, 57.8%; OR 1.98, 95% CI 1.59–2.48; *p* < 0.001), and pediatric ICU patients showed higher resistance prevalence than adult ICU patients (126/178, 70.8% vs. 1077/1768, 60.9%; OR 1.55, 95% CI 1.11–2.18; *p* = 0.010). Patient sex was not significantly associated with resistance (665/1104, 60.2% vs. 538/842, 63.9%; OR 0.86, 95% CI 0.71–1.03; *p* = 0.100). Patients with resistant isolates were slightly younger than those with susceptible isolates (median 56.6 vs. 59.0 years; *p* = 0.001), as shown in Figure 4.

### 3.9. Seven-Day Mortality by Resistance Status

Overall seven-day mortality was 43.1% (839/1946; Table 1). Mortality was 44.1% (530/1203) among patients whose first eligible isolate was 3GC-resistant and 41.6% (309/743) among those with a susceptible first isolate (unadjusted OR 1.11, 95% CI 0.92–1.33; *p* = 0.285). This comparison is unadjusted for age, sex, malignancy type, ICU setting, severity of illness or organism.

## 4. Discussion

In this decade-long analysis, 3GC resistance among Gram-negative isolates from oncology ICU patients was high: 59.2% in *Enterobacterales* and other fermenters and 67.2% in non-fermenters (assessed against fewer agents), with annual prevalence above 50% in every year. These rates are comparable with those reported in patients with cancer and in critically ill patients [7,8,9,10].”This supports the notion that cancer, especially when associated with admission to the ICU, represents a unique epidemiologic entity where community and hospital floor resistance thresholds are unlikely to apply [8,9]. Regional single-center ICU studies among oncology patients, such as the Alexandria-based study by Mohamed et al., have shown similar increases in prevalence of resistance to Gram-negative bacteria among critically ill patients with cancer [17].

*E. coli* was the most frequent isolate (30.5%), followed by *K. pneumoniae*, the *A. calcoaceticus-baumannii* complex and *P. aeruginosa*. Earlier reports in patients with cancer more often list *P. aeruginosa* and *Enterobacterales* as leading pathogens [8,9,10]. Resistance in the *A. calcoaceticus-baumannii* complex was near-universal (96.9%), consistent with its WHO critical-priority status [1,4]. This result strongly suggests that 3GCs, and quite plausibly several additional beta-lactam classes, offer negligible reliable coverage against this organism within the present institutional context, a conclusion consistent with international surveillance data describing extensive and often pan-class resistance in carbapenem-associated *Acinetobacter* lineages [1,2].

High 3GC resistance in *E. coli* and *K. pneumoniae* is in line with studies of ESBL-producing *Enterobacterales* in hematological malignancy [19,20,21,22]. We did not test for ESBL or AmpC production, so we cannot attribute our results to specific mechanisms.” *P. aeruginosa*, by contrast, exhibited a markedly lower resistance prevalence relative to the dominant *Enterobacterales* and *Acinetobacter* groups. While this organism, however, retains substantial clinical significance in oncology critical care given its inherent virulence and capacity for rapid resistance acquisition, the comparatively preserved 3GC susceptibility observed here is consistent with a recent single-center cohort study of *P. aeruginosa* bloodstream infections in hematological patients, which identified fluoroquinolone prophylaxis and prior carbapenem or piperacillin-tazobactam exposure, rather than cephalosporin exposure, as risk factors for multidrug-resistant *P. aeruginosa* [23].

The distribution of resistance was statistically significantly different based on site of isolation. Resistance prevalence varied by specimen source and was 70.0% in blood isolates. No formal test was performed, and specimen source and organism are confounded. Pleural fluid (75.8%, *n* = 33) and abdominal wall (71.1%, *n* = 45) isolates showed similarly high or higher percentages, but numbers were small. The high resistance prevalence in bloodstream isolates is concerning, as bacteremia is associated with substantial mortality and treatment failure in neutropenic and oncology ICU patients [8,9,10]. This pattern is consistent with previous reports that ESBL-producing organisms are over-represented among bloodstream infections in patients with hematological malignancy [20,21,22]. However, we did not test for ESBL production, so we cannot attribute our findings to this mechanism. Exploratory association analysis revealed several clinically relevant correlates of 3GC resistance. Resistance prevalence was notably higher among patients with hematological malignancy versus solid tumors, which is consistent with the rationale surrounding prolonged neutropenia, frequent chemotherapy exposure, recurrent hospitalization, and transplant-related exposures increasing colonization pressure/resistant infection risk in this population [8,9,19,20]. This result supports the prior literature specifically documenting elevated resistant Gram-negative infection risk among patients with hematological, as opposed to solid, malignancy [8,9,21,22,24].

Pediatric ICU patients likewise demonstrated significantly higher resistance prevalence than their adult counterparts. This finding warrants cautious interpretation, given the comparatively smaller size of the pediatric subgroup and the likelihood of a distinct underlying case mix, malignancy type distribution, and antimicrobial exposure history relative to the adult population. Nonetheless, this observation carries important practical implications, which support considering a separate pediatric oncology ICU antibiogram, rather than reliance on a consolidated, all-ages resistance summary, a recommendation consistent with established antibiogram methodology emphasizing the importance of clinically meaningful stratification [14,15].

In unadjusted analysis, seven-day mortality did not differ significantly by resistance status (44.1% vs. 41.6%; OR 1.11, 95% CI 0.92–1.33; *p* = 0.285). This should not be interpreted as showing that resistance has no clinical consequence: the analysis did not account for severity of illness, neutropenia, infection versus colonization, source control, timeliness of effective therapy or organism, and mortality was assessed only over seven days. An adjusted, episode-level analysis is warranted [18]. Strengths of this study include the extended duration of surveillance, which spanned ten years, the inclusion of adult and pediatric oncology patients treated in the ICU, and the use of standardized CLSI breakpoints during the entire study period.

Limitations include those inherent to a single-center, retrospective study. A subset of patients contributed more than one isolate (mean 1.8 per patient), and our monthly de-duplication rule was less restrictive than the CLSI M39 first-isolate convention. Isolate-level prevalence (overall, by organism, specimen source and year) may therefore be inflated by repeat isolates from persistently colonized patients. Their confidence intervals and *p*-values assume independence and are likely too optimistic. We mitigated this by re-estimating overall prevalence and all association analyses at the patient level, but we did not apply clustering-adjusted models, and the patient-level analyses reflect each patient’s first eligible isolate only. Isolates were ascertained using a laboratory-based surveillance approach analogous to the NHSN LabID Event framework rather than prospective infection-preventionist adjudication (i.e., NHSN Infection Surveillance methodology); consequently, isolates were not classified by clinical infection versus colonization status, and the reported resistance prevalence reflects all eligible isolates rather than clinically confirmed infections alone, which may over- or under-estimate the resistance burden specifically attributable to true clinical infection. Additionally, because only final categorical susceptibility results—rather than raw minimum inhibitory concentration or zone-diameter data—were retained, isolates were interpreted using the CLSI edition in effect at testing, and only categorical results were retained, so we could not reinterpret them with a single edition. CLSI breakpoints for ceftazidime and ceftriaxone in the organism groups analyzed were unchanged across the editions used (Table S1). However, changes in VITEK 2 software or card versions, or in the criteria applied to cefixime, and changes in breakpoints for comparator agents, may account for part of the year-to-year variation, which complicates interpretation of the trend.

Associations between resistance and malignancy type, ICU setting, sex and seven-day mortality were assessed using unadjusted chi-square tests and odds ratios. No multivariable model was fitted, so residual confounding, particularly by organism distribution, severity of illness, neutropenia and antimicrobial exposure, cannot be excluded. Furthermore, while the primary resistance prevalence and organism/specimen-level analyses were conducted at the isolate level, consistent with standard antibiogram reporting, the clinical and demographic association analyses (malignancy type, ICU setting, sex, and mortality) were re-derived at the patient level, using one resistance-status observation per patient, to avoid conflating isolate-level and patient-level units of analysis and to ensure mortality was assessed only once per patient; nonetheless, these patient-level associations reflect only each patient’s first eligible isolate and may not capture resistance acquired later during a patient’s clinical course. Future studies should include multivariable and episode-level adjusted analyses, treatment exposure data, and molecular characterization of isolates to better understand local resistance mechanisms and appropriately inform institutional stewardship and empirical therapy guidance.

Given the marked variation in 3GC resistance by organism and patient population, we recommend local, population-specific antibiograms to inform, rather than dictate, empirical therapy. Because treatment exposure and clinical outcomes were not evaluated, we make no recommendations on individual antibiotic agents.

## 5. Conclusions

In this ten-year cohort of oncology ICU patients, third-generation cephalosporin resistance was high overall and varied widely by organism, from near-universal in the *Acinetobacter calcoaceticus-baumannii* complex and *Pseudomonas aeruginosa*. In unadjusted patient-level analyses, resistance was more frequent in patients with hematological malignancy and in pediatric ICU patients, and was not associated with seven-day mortality. These findings support developing local, population-specific antibiograms to inform institutional antimicrobial stewardship.

## Figures and Tables

**Table 1:** Baseline demographic and clinical characteristics of the patient cohort (*n* = 1946).

Characteristic	*n*	%
Age (years)		
Mean ± SD	53.47 ± 20.07	—
Range (min–max)	1–95	—
Sex		
Male	1104	56.7%
Female	842	43.3%
Malignancy type		
Solid tumor	1440	74.0%
Hematological tumor	506	26.0%
ICU setting		
Adult ICU (AICU)	1768	90.9%
Pediatric ICU (P.ICU)	178	9.1%
Seven-day mortality		
Dead	839	43.1%
Alive	1107	56.9%
Total	1946	100%

**Table 2:** Organism distribution and third-generation cephalosporin resistance among Gram-negative isolates.

Organism	Isolates	Distribution *	Resistant **	Resistance
*Escherichia coli*	1077	30.5%	759	70.5%
*Klebsiella pneumoniae*	691	19.6%	412	59.6%
*Acinetobacter calcoaceticus baumannii* complex	607	17.2%	588	96.9%
*Pseudomonas aeruginosa*	588	16.7%	218	37.1%
*Enterobacter cloacae*	119	3.4%	78	65.5%
*Proteus mirabilis*	101	2.9%	13	12.9%
*Serratia marcescens*	64	1.8%	13	20.3%
*Klebsiella oxytoca*	37	1.0%	10	27.0%
*Stenotrophomonas maltophilia*	37	1.0%	22	59.5%
*Klebsiella aerogenes*	34	1.0%	15	44.1%
*Morganella morganii*	31	0.9%	3	9.7%
Other **	142	4.0%	56	39.4%

* Note: Proportions are isolate-level and descriptive. Repeat isolates from the same patient are not independent; no confidence intervals are shown for this reason. ** Note: Ceftriaxone and cefixime were not routinely tested against *P. aeruginosa* (0% and 0% tested, respectively) or *Acinetobacter* spp. (100% and 0% tested, respectively); 3GC resistance for these organisms therefore reflects ceftazidime susceptibility alone (*P. aeruginosa*) or ceftazidime plus ceftriaxone (*Acinetobacter* spp.), not the full three-agent panel applied to *Enterobacterales*. “Other **” comprises 26 additional Gram-negative species/groups, each individually representing <1% of isolates (e.g., *Citrobacter freundii*, *n* = 18; *Acinetobacter lwoffii*, *n* = 11; and further species/groups with *n* ≤ 8 each).

**Table 3:** Distribution and resistance rates of Gram-negative isolates by specimen source.

Specimen Source	Isolates	Resistant	Resistance
Trap	1369	805	58.8%
Urine	649	406	62.6%
Blood	406	284	70.0%
Abdomen	107	65	60.7%
Peritoneal Fluid	95	61	64.2%
Bronchial Lavage	74	47	63.5%
Sacral Region	67	40	59.7%
Sputum	55	29	52.7%
Abdominal Wall	45	32	71.1%
Pleural Fluid	33	25	75.8%
Bile Secretion	32	16	50.0%
Wound	31	19	61.3%
Other *	565	358	63.4%

* “Other” comprises 111 additional specimen source categories, each individually representing <1% of isolates (e.g., neck, *n* = 38; back, *n* = 31; buttock; and further categories with *n* ≤ 13 each).

**Table 4:** Susceptibility patterns of Gram-negative isolates to non-cephalosporin and comparator antimicrobial agents.

Agent	Tested	Resistance	Susceptible
Piperacillin–tazobactam	3463	35.5%	55.0%
Ciprofloxacin	3454	58.1%	35.8%
Gentamicin	3427	37.3%	61.1%
Amikacin	3434	24.7%	72.9%
Imipenem	3189	26.7%	72.8%
Meropenem	3213	26.3%	73.3%
Ertapenem	2216	12.7%	86.4%

## Data Availability

De-identified data are available from the corresponding author upon reasonable request, subject to KHCC IRB approval.

## Supplementary Materials

The following supporting information can be downloaded at: https://www.mdpi.com/article/10.3390/pathogens15101083/s1, Table S1: CLSI M100 edition and VITEK 2 configuration applied to antimicrobial susceptibility interpretation, by study year (2015–2024); Table S1 (continued): Third-generation cephalosporin MIC breakpoints applied, by organism group. Ceftazidime and ceftriaxone breakpoints for the organism groups analyzed were identical in all editions used (2015–2024).

## Abbreviations

The following abbreviations are used in this manuscript:

3GC(s)	Third-generation cephalosporin(s)
AMR	Antimicrobial resistance
CDC	Centers for Disease Control and Prevention
CI	Confidence interval
CLSI	Clinical and Laboratory Standards Institute
ESBL	Extended-spectrum beta-lactamase
ICU	Intensive care unit
IQR	Interquartile range
IRB	Institutional Review Board
KHCC	King Hussein Cancer Center
MDRO	Multidrug-resistant organism
OR	Odds ratio
SD	Standard deviation
WHO	World Health Organization
