# Supplementary material for: Characteristics and Resistance Patterns of Gram-Negative Bacterial Isolates to Third-Generation Cephalosporins in Oncology ICU Patients: A Ten-Year Retrospective Analysis
Source: Pathogens. 2026 Oct 15;15(10):1083. doi: 10.3390/pathogens15101083 (PMC13708355; doi:10.3390/pathogens15101083)
Supplement: Supplementary file 1 [file pathogens-15-01083-s001.zip › pathogens-4549617-supplementary.pdf]

Table S1. CLSI M100 edition and VITEK 2 configuration applied to antimicrobial susceptibility interpretation, by study year (2015–2024).

Year	CLSI M100 edition applied	Edition published	Change in ceftazidime and ceftriaxone breakpoints vs. previous edition
2015	M100-S25	2015	Reference edition
2016	M100-S26	2016	None
2017	M100-S27	2017	None
2018	M100-S28	2018	None
2019	M100, 29th ed.	2019	None
2020	M100, 30th ed.	2020	None
2021	M100, 31st ed.	2021	None
2022	M100, 32nd ed.	2022	None
2023	M100, 33rd ed.	2023	None
2024	M100, 34th ed.	2024	None

Isolates were interpreted with the M100 edition in effect at the time of testing, as applied by the VITEK 2 expert system. Only final categorical results (S/I/R) were retained, and isolates were not reinterpreted using a single current edition.

Table S1 (continued). Third-generation cephalosporin MIC breakpoints applied, by organism group. Ceftazidime and ceftriaxone breakpoints for the organism groups analyzed were identical in all editions used (2015–2024).

Organism group	Ceftazidime MIC (μg/mL)	Ceftriaxone MIC (μg/mL)
Enterobacterales and other fermenters	S ≤4; I 8; R ≥16	S ≤1; I 2; R ≥4
Pseudomonas aeruginosa	S ≤8; I 16; R ≥32	Not tested (no CLSI breakpoints)
Acinetobacter spp.	S ≤8; I 16; R ≥32	S ≤8; I 16–32; R ≥64

3GC, third-generation cephalosporin; AST, antimicrobial susceptibility testing; CLSI, Clinical and Laboratory Standards Institute; MIC, minimum inhibitory concentration; S/I/R, susceptible/intermediate/resistant. Ceftriaxone and cefixime were not tested against P. aeruginosa, and cefixime was not tested against Acinetobacter spp. (see Methods).
